# Prediction of rehospitalization in patients with acute heart failure using point-of-care lung ultrasound

**DOI:** 10.1186/s12872-022-02781-9

**Published:** 2022-07-24

**Authors:** I. Rattarasarn, T. Yingchoncharoen, T. Assavapokee

**Affiliations:** 1 Department of Medicine, Faculty of Medicine, Ramathibodi Hospital, Mahidol University, Rama VI Road, Thung Phaya Thai, Ratchathewi, Bangkok, 10400 Thailand; 2 Division of Cardiology, Department of Medicine, Faculty of Medicine, Ramathibodi Hospital, Mahidol University, Rama VI Road, Thung Phaya Thai, Ratchathewi, Bangkok, 10400 Thailand; 3 Division of Geriatric Medicine, Department of Medicine, Faculty of Medicine, Ramathibodi Hospital, Mahidol University, Rama VI Road, Thung Phaya Thai, Ratchathewi, Bangkok, 10400 Thailand

**Keywords:** B-line, Point-of-care ultrasound, Heart failure, Rehospitalization

## Abstract

**Background:**

More than 50% of patients admitted for acute heart failure are discharged with residual pulmonary congestion. Residual pulmonary congestion at discharge is associated with rehospitalization and death within 6 months after discharge. B-lines detected by lung ultrasound are the sonographic manifestation of pulmonary congestion, a major predictor of morbidity and mortality in patients with heart failure. The present study was performed to evaluate the prognostic value of B-lines at discharge for prediction of rehospitalization and death at 6 months in patients with acute heart failure.

**Methods:**

This study involved a prospective cohort of 126 patients admitted to Ramathibodi Hospital for acute heart failure (mean age, 69 ± 15 years). B-lines and the size of the inferior vena cava were assessed within 24 h before discharge. The patients were followed up for 6 months after discharge.

**Results:**

The mean number of B-lines at discharge was 9 ± 9, and the rate of rehospitalization within 6 months was significantly higher in patients with a significant number of B-lines (≥ 12) than in patients with a non-significant number of B-lines (< 12) (log rank χ^2^ = 7.74, *P* = 0.004). In the univariable analysis, the presence of ≥ 12 B-lines before discharge (hazard ratio = 2.15, 95% confidence interval = 1.27–3.63) was an independent predictor of events at 6 months.

**Conclusions:**

Residual pulmonary congestion before discharge as detected by point-of-care lung ultrasound predicts rehospitalization for heart failure at 6 months. The presence of non-significant B-lines identifies a subgroup at low risk of rehospitalization for heart failure.

**Supplementary Information:**

The online version contains supplementary material available at 10.1186/s12872-022-02781-9.

## Introduction

Heart failure (HF) is a major health burden in Thailand. The prevalence of symptomatic HF in Thailand is estimated to range from 0.4 to 2.0% in the general population. The most common precipitating cause of HF is heart disease itself, and one study showed that 54% and 20% of cases of HF were related to inadequate diuretics and poor patient compliance with medications, respectively [1].

Patients with HF often develop congestion that may require urgent hospitalization. A significant proportion of patients with acute HF are discharged with persistent congestion, which is associated with a higher risk of readmission and mortality [2]. Residual pulmonary congestion should be assessed before discharge. However, congestion can be difficult to assess, especially when extrapulmonary signs of congestion are mild. Clinical scores that combine several clinical indicators have been shown to assess the level of congestion more accurately than physical assessment alone. Many prognostic scores are available, but the most evidence-based score in the current era of acute HF management is the EVEREST score [3].

Based on current guidelines, lung ultrasound may be considered in the assessment of pulmonary congestion [2]. A B-line is defined as a discrete laser-like vertical hyperechoic reverberation artifact that arises from the pleural line, extends to the bottom of the screen without fading, and moves synchronously with lung sliding. Significant B-lines are defined as the presence of three or more B-lines in a longitudinal plane between two ribs and two or more positive regions in each lung, and their presence suggests significant pulmonary congestion [4]. If persistent pulmonary congestion is present, the treatment, including an increased diuretic dose, should be optimized to keep the patient free of congestion [2].

## Methods

### Study design

This single-center observational prospective cohort study involved 130 adult patients prospectively enrolled in Ramathibodi Hospital after their index hospitalization for a primary diagnosis of acute HF according to the Framingham criteria [5] from July 2020 to July 2021. Of these 130 patients, 4 did not satisfy the inclusion criteria because of the initiation of renal replacement therapy after discharge. Therefore, 126 patients were enrolled in the study.

The inclusion criteria were (1) an age of ≥ 18 years, (2) acute HF diagnosed by the Framingham criteria, and (3) the ability to provide informed consent. The exclusion criteria were (1) end-stage renal disease or treatment with renal replacement therapy; (2) isolated right-sided HF; (3) pulmonary disease affecting lung ultrasound interpretation, such as interstitial lung disease, pneumonia, or pleural disease; and (4) pregnancy. The treatment course and discharge decisions were dependent upon the cardiologist service team. Physical examination, lung ultrasound, and echocardiography were performed within 24 h before discharge. The follow-up data were obtained at 6 months by medical record reviews and telephone interviews.

**Fig. 1:**
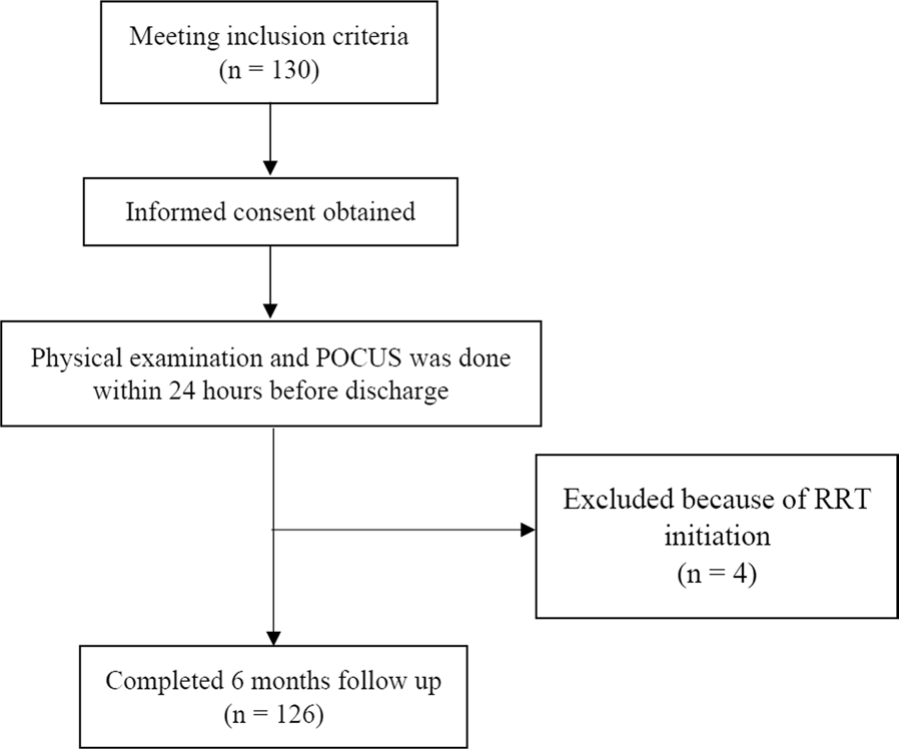
Study design flow chart. *RRT* renal replacement therapy

The study design flow chart is shown in Fig. 1.

### Study outcomes

The primary outcome was the association between the number of B-lines and HF events or all-cause mortality within 180 days. An HF event was defined as an urgent HF visit and hospitalization for HF. An urgent HF visit was defined as an unscheduled visit to the outpatient department or emergency department as a result of signs and/or symptoms of worsening HF that required intravenous diuretic treatment with a hospital stay of < 1 day, and hospitalization for HF was defined as a stay traversing a change in calendar date (> 1 day).

The secondary outcomes were the association between the EVEREST score and HF events or all-cause mortality within 6 months and the association between the number of B-lines and the right atrial pressure (RAP).

### Lung ultrasound and echocardiographic study

The patients underwent lung ultrasound and echocardiography by a trained examiner within 24 h before discharge. Comprehensive lung ultrasound and echocardiography were performed with a Lumify S4-1 phased array transducer (Philips Healthcare, Amsterdam, The Netherlands), if available; otherwise, a phased array or convex transducer was used.

Lung ultrasound examinations were performed by using a simplified 8-zone imaging protocol (4 zones on each hemithorax) in the sagittal orientation at an imaging depth of 16 cm, with the patients in the semi-recumbent position [4].

RAP was typically estimated based on the dimensions of the inferior vena cava (IVC). Measurements of the IVC were obtained with the patient in the supine position in the sub-xyphoid transabdominal long axis, 2–3 cm caudal to the right atrial junction, and the IVC diameter was measured in motion-mode (M-mode) [6]. A high maximum IVC diameter (≥ 21 mm) indicated hemodynamic congestion. The degree of inspiratory collapse of the distal tract of the IVC was also examined. The discriminating value for diagnosis of IVC collapse was an IVC collapsibility index (IVCCI) of 50%. An IVCCI of < 50% was assumed to indicate hemodynamic congestion [7].

The intraobserver and interobserver reliability of B-line quantification and the IVC diameter have been a previously tested and calculated for agreement by Cohen’s kappa statistic. Good interobserver agreement was assumed in these tests when the coefficient values were ≥ 0.75 [8].

### Statistical analysis

Continuous variables are expressed as mean ± standard deviation. HF events and all-cause mortality were examined using Kaplan–Meier analysis and compared using the log-rank test. Event and death rates were estimated with Kaplan–Meier curves and compared by the log-rank test. Cox proportional hazards regression modeling was used to examine the association between number of B-lines and HF events or all-cause mortality at 6 months, using univariable and stepwise multivariable procedures. A *P* value of < 0.05 was considered statistically significant. Statistical analysis was performed using STATA version 17.0 (StataCorp, College Station, TX, USA).

## Results

### Patient characteristics

The baseline characteristics of the final study population of 126 patients are shown in Table 1. In the whole cohort, 53.2% of patients were male, and their mean age was 69 ± 15 years. The most common comorbidities were hypertension (69.1%), diabetes mellitus (48.4%), and previous HF of any cause (45.2%). At admission, 88.1% of patients presented with dyspnea on exertion, and rales were detected by lung examination in 87.3% of patients. Cardiomegaly was found on chest radiographs of all patients, and cephalization and pleural effusion were found in 92.1% and 51.6%, respectively. The main cause of acute HF was acute coronary syndrome in 38 (30.2%) patients and inadequate diuresis and/or poor compliance in 19 (13.5%). A total of 48 (38.1%) patients had a left ventricular ejection fraction (LVEF) of ≥ 50%, and 78 (61.9%) had an LVEF of < 50%.

**Table 1:** Clinical and demographic characteristics of patients with acute HF (N = 126)

Age, years	69 ± 15
Male	67 (53.17)
*Medical history*	
Hypertension	87 (69.05)
Diabetes mellitus	61 (48.41)
Hypercholesterolemia	91 (72.22)
Coronary artery disease	38 (30.16)
Arrhythmia	41 (32.54)
Previous HF	57 (45.24)
*Clinical*	
PND/orthopnea	78 (61.90)
Peripheral edema	87 (69.04)
Dyspnea on exertion	111 (88.10)
Nocturnal cough	15 (11.90)
Rales	110 (87.30)
Heart rate of > 120 beats/min	12 (9.52)
*Hemodynamic profile*	
Wet and warm	115 (91.27)
Wet and cold	9 (7.14)
Dry and warm	2 (1.59)
Dry and cold	0 (0)
*Admission chest radiograph findings*	
Cardiomegaly	126 (100)
Cephalization	111 (92.06)
Pleural effusion	65 (51.59)
*Precipitating causes*	
Acute coronary syndrome	38 (30.16)
Arrhythmia	15 (11.90)
Valvular heart disease	8 (6.35)
Infection	7 (5.56)
Cardiomyopathy	15 (11.90)
Hypertensive emergency	10 (7.93)
Pulmonary hypertension	3 (2.38)
Anemia	8 (6.35)
Inadequate diuresis or poor compliance	19 (13.49)
Other	5 (3.97)
*Baseline laboratory results*	
HbA1c, %	6.38 ± 1.27
LDL, mg/dL	97.24 ± 46.43
BUN, mg/dL	26.19 ± 15.66
Cr, mg/dL	1.56 ± 1.04
GFR	54.59 ± 27.47
ALT, IU/L (n = 109)	24.00
GGT, IU/L (n = 108)	60.00
Total bilirubin, IU/L (n = 108)	1.24 ± 1.08
Troponin T, ng/dL (n = 110)	58.55
NT pro-BNP, pg/mL (n = 101)	6,230
*LVEF, %*	
≥ 50%	48 (38.09)
40–49%	20 (15.88)
< 40%	58 (46.03)
*EVEREST score at discharge*	
≥ 1 point	92 (72.65)
≥ 3 points	17 (13.28)

Data are presented as mean ± standard deviation, n (%), or median value

*HF* heart failure, *PND* paroxysmal nocturnal dyspnea, *HbA1c* glycated hemoglobin, *LDL* low-density lipoprotein, *BUN* blood urea nitrogen, *Cr* creatinine, *GFR* glomerular filtration rate, *ALT* alanine aminotransferase, *GGT* gamma-glutamyl transpeptidase, *NT pro-BNP* N-terminal B-type natriuretic peptide, *LVEF* left ventricular ejection fraction

Intraobserver and interobserver reliability for the B-line quantity and IVC diameter was calculated by Cohen’s kappa statistic for agreement (Additional file 1: Table S1 in the supplementary appendix). About 72.7% of patients were discharged with an EVEREST score of ≥ 1 point. The number of B-lines at discharge ranged from 0 to 18, and 32.54% of patients had residual pulmonary congestion. The main medications at discharge were beta-blockers (76.9%), furosemide (71.2%), and spironolactone (31.8%) (Table 2). Only 25.9% of patients who had HF with a reduced ejection fraction received optimized guideline-directed medical therapy (GDMT). The main reasons for not using an angiotensin-converting enzyme inhibitor (ACEI) or angiotensin receptor blocker (ARB) were HF with a preserved ejection fraction, acute kidney injury, and chronic renal failure.

**Table 2:** HF events and precipitating causes (N = 126)

No HF events	69 (54.76)
*HF events (first episode after discharge)*	
Urgent HF visits	12 (9.52)
Hospitalization for HF	34 (26.98)
Death (all-cause mortality)	11 (8.74)
*Precipitating causes of HF*	
ACS	1 (2.17)
Arrhythmia	4 (8.70)
Infection	8 (17.39)
Hypertensive emergency	1 (2.17)
Inadequate diuresis	12 (26.09)
Salt and water retention	13 (28.26)
Poor compliance	4 (8.70)
Other	3 (6.52)

Data are presented as n (%)

*HF* heart failure, *ACS* acute coronary syndrome

Lists of medications used and dosage at discharge are shown in Additional file 1: Tables S2 and S3.

Beta-blocker is the most prescribed medication (76.89%) while the other GDMT such as ACEI/ARB was used in 38.1% and mineralocorticoid receptor antagonists in 31.75% of the patients. SGLT2 inhibitors, the latest GDMT according to the 2021 European Society of Cardiology Guidelines [2], were used 3.17%.

Empagliflozin was the only medication that patients received at the target dose before discharge.

### Follow-up data: HF events and mortality

At the 6-month follow-up, combined events occurred in 57 patients (16 urgent HF visits, 32 hospitalizations for HF, and 9 all-cause deaths). Details are shown in Table 2. The most common causes of acute HF events were salt and water retention (28.3%) and inadequate diuresis (26.1%).

Predictors of HF events and all-cause death by univariate and multivariate analyses are shown in Tables 3 and 4.

**Table 3:** Univariable analysis of 6-month HF events

Variables	HR	95% CI	*P* value
Age Male DM Hypertension Arrhythmia CCS Prior HF HbA1c level LDL level BUN level Cr level ALT level GGT level Total bilirubin level Troponin T level LVEF of < 40% EVEREST score of ≥ 1 ≥ 12 B-lines IVC diameter of > 2.1 cm IVC collapsibility of < 50% RAP Optimal GDMT	1.01 0.98 0.62 0.82 1.35 0.83 0.84 1.05 1.00 1.01 1.35 1.00 0.99 1.01 1.00 0.69 2.08 2.15 1.08 0.99 1.03 0.40	0.58–1.66 0.48–1.50 0.36–1.06 0.45–1.49 0.77–2.38 0.44–1.53 0.48–1.48 0.85–1.30 0.99–1.00 0.99–1.02 1.08–1.69 0.99–1.00 0.99–1.00 0.79–1.31 0.99–1.00 0.37–1.31 1.02–4.25 1.27–3.62 0.55–2.11 0.98–1.00 0.97–1.09 0.12–1.28	0.96 0.57 0.82 0.52 0.30 0.55 0.55 0.66 0.38 0.127 0.009 0.31 0.93 0.91 0.98 0.26 0.04 0.004 0.82 0.29 0.36 0.12

*HF* heart failure, *HR* hazard ratio, *CI* confidence interval, *DM* diabetes mellitus, *CCS* chronic coronary syndrome, *HbA1c* glycated hemoglobin, *LDL* low-density lipoprotein, *BUN* blood urea nitrogen, *Cr* creatinine, *ALT* alanine aminotransferase, *GGT* gamma-glutamyl transpeptidase, *LVEF* left ventricular ejection fraction, *IVC* inferior vena cava, *RAP* right atrial pressure, *GDMT* guideline-directed medical therapy

**Table 4:** Multivariable analysis of 6-month HF events

Variables	HR	95% CI	*P* value
Cr level EVEREST score of ≥ 1 ≥ 12 B-lines	1.30 1.77 1.96	1.04–1.63 0.86–3.65 1.14–3.37	0.02 0.12 0.02

*Cr* creatinine, *HF* heart failure, *HR* hazard ratio, *CI* confidence interval

**Fig. 2:**
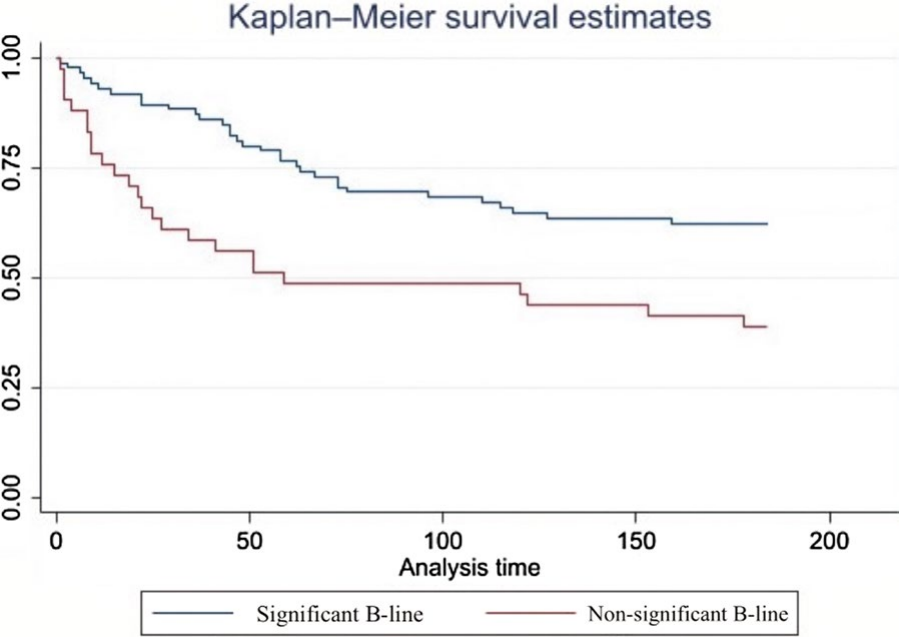
Kaplan–Meier analysis of B-line quantity, heart failure events, and all-cause death

The number of B-lines using the eight-zone method was correlated with HF events and all-cause death at 6 months (hazard ratio = 2.15, 95% confidence interval = 1.27–3.63). Kaplan–Meier curves depicting the prognostic value of significant B-lines before discharge at the 6-month follow-up are shown in Fig. 2 (log rank χ^2^ = 7.74, *P* = 0.004) that is provided in separate file. The median time of HF events was 50 days after discharge (range, 1–159 days after discharge). Loop diuretic adjustments after discharge was not associated with a difference in HF events and all-cause death in both groups (chi-square test = 1.02, *P* = 0.60) and optimal GDMT modification in HFrEF patients at 6 months was not correlated with HF events and all-cause mortality as well (chi-square test = 0.80, *P* = 0.37).

### Relationship between IVC diameter and B-line quantity

About 56.4% of patients had a low RAP and B-line quantity, and 7.15% had a high RAP and B-line quantity. Discordant results between the estimated RAP and B-line quantity were found in 36.5% of patients.

## Discussion

Clinical congestion at discharge is a strong predictor of poor outcome in patients with acute decompensated HF [9]. Therefore, patients hospitalized with HF should be thoroughly examined for signs of congestion to optimize the use of medication before discharge. Unfortunately, congestion can be difficult to assess in some patients because of the dissociation between its clinical findings and cardiac filling pressure [9]. Although there are many tools proposed for clinical implementation in current practice, standard tools to predict HF outcome are not available. Most scores use signs and symptoms of congestion as predictors. To our knowledge, this is the first prospective observational study to date that shows the prediction of rehospitalization for HF events and all-cause mortality at 6 months in patients detected to have significant pre-discharge B-lines using point-of-care lung ultrasound in the Thai population.

In the present prospective observational study, we found that hospitalized patients with HF who had ≥ 12 pre-discharge B-lines by the eight-zone method using point-of-care lung ultrasound had significantly increased risk of HF events and all-cause death at 6 months (hazard ratio = 1.96, 95% confidence interval = 1.14–3.37). Gargani et al. [10] reported that ≥ 15 significant B-lines at discharge strongly predicts rehospitalization for acute HF and death at 6 months (hazard ratio = 11.17, 95% confidence interval = 1.30–106.16) while the patients without significant B-lines at discharge had a significantly better outcome. Similar to our study, the multivariable analysis showed that the presence of significant B-lines was an independent predictor of HF events and death at 6 months. The findings of the present study encourage pre-discharge evaluation of residual pulmonary congestion using point-of-care lung ultrasound as stated by the 2021 ESC guidelines [2].

As stated by the EVEREST trial, there is a 10% increase in the probability of hospitalization at 6 months in patients with an EVEREST score of ≥ 1 point. In the present study, an EVEREST score of ≥ 1 point increased the probability of HF events by 2.08 times compared with an EVEREST score of 0 points (hazard ratio = 2.08, 95% confidence interval = 1.02–4.05). However, the presence of significant B-lines was the only predictive factor of HF events and all-cause death at 6 months as shown by the multivariate analysis.

With respect to the secondary endpoint of the study, discordance between the estimated RAP and B-line quantity was found in one-third of patients. This is in concordance with the findings from a previous study [11] that analyzed the ESCAPE trial database and found that 25–30% of advanced HF patients had right- and left-sided ventricular filling pressure mismatch. The main pathophysiology of pulmonary congestion is the increased LV filling pressure, detectable by the number of B-lines from eight-zone method by point-of-care lung ultrasound and RAP, can be assessed using IVC diameter measurement and its collapsibility. Therefore, the point-of-care ultrasound can be used in clinical practice to improve the sensitivity of congestion detection.

Because the data were collected between July 2020 and July 2021, GDMT based on the current guidelines consist of a beta-blocker, ACEI or ARB, and mineralocorticoid receptor agonist [12]. Only 25% of patients who had HF with a reduced ejection fraction received optimal GDMT. The main reasons for not using an ACEI or ARB were HF with a preserved ejection fraction, acute kidney injury, and chronic renal failure resulting in the use of nitrates and hydralazine. The 2021 ESC guidelines recommended that the GDMT should be uptitrated to the doses used in clinical trials or maximally tolerated doses, while most of our patients received GDMT at doses lower than the target ones. Although our study showed that the patients with optimized GDMT had a decreased risk of HF events and all-cause mortality at 6 months, the difference was not statistically significant.

The baseline laboratory tests showed that many patients had multiple organ injury such as acute kidney injury, or liver congestion. Previous studies [13, 14] reported associations between organ failure and risk of poor outcome. In the present study, the presence of elevated creatinine (hazard ratio = 1.30, 95% confidence interval = 1.04–1.63) at admission also correlated with rehospitalization for HF and all-cause mortality at 6 months, while ALT, GGT, total bilirubin, and cardiac troponin did not correlate with poor outcomes. In contrast to our study, Zymliński et al. [13] reported that patients with ≥ 2 organ failures (including heart, kidney, and liver) have a higher risk of poor outcome while there was no difference in patients with no or single organ injury.

This study had two main limitations. First, there were some missing data, such as percentage of change in body weight during admission, because our study was observational in nature and there was not a designated HF treatment protocol in our setting. Second, there was a limitation in the number of hospital beds. The fact that some patients were hospitalized in the emergency department might have led to inadequate diuresis before discharge. Nonetheless, this study’s results should emphasize the need for a point-of-care lung ultrasound-guided treatment protocol to be implemented in the care of acute HF patients before discharge, and should warrant future studies that compare clinical outcomes between patients who receive clinical-guided and ultrasound-guided treatment in large multicenter settings.

## Conclusion

Residual pulmonary congestion before discharge as detected by ≥ 12 B-lines on point-of-care lung ultrasound predicts rehospitalization for HF events and all-cause death at 6 months. The number of B-lines can be useful not only for the differential diagnosis of acute dyspnea but also for the prognostic stratification of patients with HF.

## Supplementary Information

**Additional file 1:** Supplemental methods, Sample size calculation, Supplementary tables. **Table S1.** Intraobserver and interobserver reliability. **Table S2.** Medication used at discharge. **Table S3.** Medication dosage at discharge.

## Data Availability

The data supporting the findings of this study are available within the article and its Additional file 1.
