# Supplementary material for: Prediction of rehospitalization in patients with acute heart failure using point-of-care lung ultrasound
Source: BMC Cardiovasc Disord. 2022 Jul 24;22:330. doi: 10.1186/s12872-022-02781-9 (PMC9310393; doi:10.1186/s12872-022-02781-9)
Supplement: Supplementary file 1 — Additional file 1: Supplemental methods, Sample size calculation, Supplementary tables. Table S1. Intraobserver and interobserver reliability. Table S2. Medication used at discharge. Table S3. Medication dosage at discharge. [file 12872_2022_2781_MOESM1_ESM.docx]

**Supplementary Appendix**

**Contents**

Supplemental methods

Sample size calculation……………………………………………………………………….2

Supplementary tables

Table S1. Intraobserver and interobserver reliability…………………………………………3

Table S2. Medication used at discharge……………………….……………………………..3

Table S3. Medication dosage at discharge……………….……..……………………………..4

Sample size calculation

Sample size is calculated by Fleiss formula^1^ and calculated sample size is 126 patients.


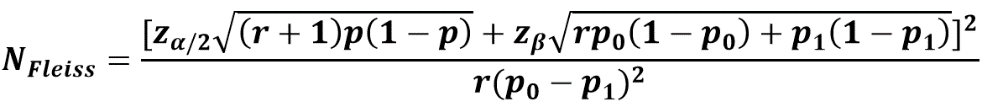


Reference

Fleiss JL, Levin B and Paik MC. Determining Sample Sizes Needed to Detect a Difference between Two Proportion. In: Fleiss JL, Levin B and Paik MC. Statistical Methods for Rates and Proportions, Third Edition. New Jersey: John Wiley & Sons; 2003. p. 64-85.

Table S1. Intraobserver and interobserver reliability

|  | Kappa |
| --- | --- |
| Intraobserver reliability (examinator 1)  B-line quantity from lung ultrasound  IVC diameter assessment  Intraobserver reliability (examinator 2)  B-line quantity from lung ultrasound  IVC diameter assessment | 1.0000  0.9230  1.0000  0.9230 |
| Interobserver reliability  B-line quantity from lung ultrasound  IVC diameter assessment | 0.7727  0.8276 |

Table S2. Medications used at discharge (N = 126)

| Furosemide  Hydralazine  Nitrate  ACEI/ARB  Beta-blocker  Mineralocorticoid receptor  Sacubitril/valsartan  Ivabradine  Tolvaptan  Digoxin  SGLT2 inhibitor | 91 (71.22)  33 (26.19)  37 (29.37)  48 (38.10)  97 (76.89)  40 (31.75)  8 (6.35)  7 (5.56)  3 (2.38)  9 (7.14)  4 (3.17) |
| --- | --- |

Data are presented as n (%).

ACEI, angiotensin-converting enzyme inhibitor; ARB, angiotensin receptor blocker; SGLT2, sodium-glucose cotransporter 2

Table S3. Medications used at discharge (N = 126)

| Furosemide, mg/day  Hydralazine, mg/day  Nitrate  Isosorbide dinitrate, mg/day  Isosorbide mononitrate, mg/day  ACEI/ARB  Enalapril, mg/day  Losartan, mg/day  Valsartan, mg/day  Candesartan, mg/day  Azilsartan, mg/day  Beta-blocker  Bisoprolol, mg/day  Carvedilol, mg/day  Nebivolol, mg/day  Metoprolol, mg/day  Propanolol, mg/day  Spironolactone, mg/day  Sacubitril/valsartan, mg/day  Ivabradine, mg/day  Tolvaptan, mg/day  Digoxin, mcg/day  SGLT2 inhibitor  Empaglifozin, mg/day | 40  100  30  40  10  50  80  8  40  2.5  12.5  5  75  20  25  49/51  10  7.5  125  10 |
| --- | --- |

Data are presented as median value.

ACEI, angiotensin-converting enzyme inhibitor; ARB, angiotensin receptor blocker; SGLT2, sodium-glucose cotransporter 2
